# Evaluation of Immune Response Dynamics: Analyzing the Parameters of Complete Blood Count (CBC) in Experimental Borreliosis

**DOI:** 10.3390/life15111758

**Published:** 2025-11-16

**Authors:** Diana Mihaela Alexandru, Diana Larisa Ancuţa, Cristin Coman

**Affiliations:** 1 Faculty of Veterinary Medicine, University of Agronomic Sciences and Veterinary Medicine, 050097 Bucharest, Romania; 2 Cantacuzino National Military-Medical Institute for Research and Development, 050096 Bucharest, Romania; diana.larisa.ancuta@gmail.com; 3 Center of Excellence in Translational Medicine, Fundeni Clinical Institute, 022328 Bucharest, Romania

**Keywords:** *Borrelia bavariensis*, neutrophil–lymphocyte ratio, systemic immune inflammatory index, murine model, Lyme disease

## Abstract

The spirochete *Borrelia* is responsible for Lyme disease, a multisystemic infection and growing public health concern. This study aimed to evaluate host response dynamics to *Borrelia bavariensis* by analyzing hematological parameters as potential immuno-inflammatory markers in a murine model. Forty C3He/HeNCrl mice were inoculated intradermally with *B. bavariensis* (5 × 10^5^ spirochetes/100 µL/mouse) and monitored for 90 days. Samples were collected at defined intervals for microbiological examination, hematology, and qPCR. Microbiological and qPCR testing revealed infection between days 7–21; results were negative on days 28–42. At later stages (days 60 and 90), *Borrelia* was only detectable by qPCR, highlighting differences in diagnostic sensitivity. Hematological analysis showed that the neutrophil-to-lymphocyte ratio (NLR) and systemic immuno-inflammatory index (SII) peaked on day 7 (*p* < 0.0001), followed by gradual normalization until day 35. These markers reflected the intensity of the inflammatory response and defined three distinct phases of host reaction. Overall, results demonstrate the complexity of immune responses in *B. bavariensis* infection and underscore the value of monitoring hematological indices for understanding host–pathogen interactions. This approach supports the potential use of simple blood markers in diagnostic strategies with translational relevance for clinical practice.

## 1. Introduction

The spirochete *Borrelia bavariensis* is a causative agent of Lyme disease (LD), an infectious disease with multisystemic effects that has become a major public health concern [1,2]. Globally, LD is currently the most common tick-borne disease [3], with an estimated number of over 450,000 diagnosed cases in the United States and over 200,000 cases in Europe [4,5]. LD was considered a rare and localized condition, or less known and diagnosed, and its incidence has increased significantly [2]. This increase requires a deeper understanding of pathogenesis, especially all immunological processes that occur in infected patients. Lyme borreliosis is transmitted by ticks belonging to the genus *Ixodes* (in Western Europe *Ixodes ricinus*) that have been infected with spirochetes from the *Borrelia burgdorferi sensu lato* complex (Bbsl) [6]. Phylogenetic analyses have currently identified 24 genospecies; we must add *Borrelia maritima* [7] to the 23 already reported [8], and soon we will probably also add a 25th, candidatus *Borrelia sibirica* [9].

*Borrelia bavariensis* is one of its members, with tropism for the nervous system [10,11]. LD affects multiple organs, showing tropism for the joints (*Borrelia burgdorferi sensu strico*—Bbss), skin (*Borrelia afzelii* and *Borrelia valaisiana*), eyes, and heart. *Borrelia bavarensis* and *Borrelia garinii* have tropism mainly for the nervous system and can lead to debilitating and persistent symptoms [12]. Although antibiotics serve as standard treatment, relapses are observed especially when diagnosis and/or treatment is late. Post Treatment Lyme Disease Syndrome (PTLDS) may be due to the survival of persistent forms of spirochetes after treatment [1], or to immune mechanisms and it is often difficult to distinguish these forms with serological testing. Probably in the near future Proteomics will allow us to identify cases of PTLD in which Bbsl is still alive [13]. *Borrelia* species evade the host immune system by binding factor H and altering surface proteins, allowing them to persist in the bloodstream and spread to immune-privileged sites [6,14], while spirochaetemic persistence in humans is observed only for *Borrelia mayonii* [15]. This leads to early flu-like symptoms and skin lesions, progressing to more severe complications like arthritis and neurological issues. Chronic infection is driven by immune evasion and sustained inflammation, with excessive IFN-γ activity linked to impaired tissue healing [16,17]. In addition, several studies have highlighted the role of type I interferons (IFN-α/β) in the pathogenesis of chronic Lyme borreliosis, showing that their sustained activation can contribute to immune dysregulation, inhibition of effective bacterial clearance, and promotion of persistent inflammation [18]. The type I IFN response, typically antiviral, may paradoxically favor spirochete persistence by modulating macrophage activation and altering cytokine signaling pathways, thus delaying resolution of the infection [19].

Experimental studies on animal models, particularly monkeys and dogs, indicate that *Borrelia* spirochetes localize extensively within the nervous system (both peripherally and centrally) [20]. The pathological mechanisms remain unclear, but the presence of the spirochetes DNA in neural tissues and the associated tissue damage point to a complex interplay of immune response and potential neurotoxic effects [12]. In humans, the formation of biofilm also protects Bbsl from the immune system and antibiotics [21]. The mouse model of LD demonstrates an immune response similar to that seen in certain patients; however, it does not present a wide range of other symptoms associated with the disease [12]. C3H/HeN mice showed severe neutrophilic infiltration in periarticular structures, peaking weeks post-infection, while *B. burgdorferi*-infected C57BL/6 mice show little to no arthritis [17]. Immunodeficient RAG-/- and SCID mice on a C57BL/6 background resist arthritis, suggesting factors beyond humoral and cellular immunity influence disease severity [22]. These results indicate strain-dependent arthritis severity, with neutrophils, macrophages, and IFN-γ/NF-κB signaling driving inflammation, similar to human Lyme arthritis [17].

In their research, Barthold et al. (1991) found that, in mice infected with *Borrelia*, joint and tendon inflammation appeared between days 4–7, followed by heart inflammation from days 7–10, coinciding with tissue colonization by spirochetes in collagen-rich areas. IgM antibodies were detectable by day 4 but declined over time, while IgG antibodies appeared by day 7 and increased through day 30 [23]. Further research by Barthold and Bockenstedt (1993) showed that early protective antibodies develop independently of outer surface protein A, and their decline over time suggests antigenic changes or immune suppression despite ongoing infection [24].

Complete blood count (CBC) parameters are accessible and cost-effective biomarkers that can reflect inflammatory and immune responses during various infectious and inflammatory processes [25,26]. Recently, predictive models based on ratios between CBC components have proven valuable in the diagnosis and monitoring of infectious diseases such as COVID-19, sepsis [27], and other bacterial infections [28]. The CBC has been evaluated as an auxiliary tool in the diagnosis and monitoring of Lyme borreliosis [29]. Although hematological changes are not specific for this infection, multiple studies have demonstrated that there may be alterations in blood parameters that, in conjunction with clinical manifestations, can guide the diagnosis [29,30]. In one study, it was highlighted that patients with neuroborreliosis may present changes in the leukocyte formula, including mild lymphocytosis in the cerebrospinal fluid, which may have diagnostic value in correlation with clinical manifestations [31].

Aguero-Rosenfeld et al. (2005) emphasized in their comprehensive analysis of laboratory tests in borreliosis that, although the complete blood count is not specific for diagnosis, it may reflect the systemic inflammatory response in the different stages of the disease [32]. Other researchers have reported that patients with erythema migrans may present with mild thrombocytopenia and changes in the white blood cell count, which may be correlated with the severity of clinical manifestations and response to treatment [33]. In a prospective analysis of hematological parameters in patients with confirmed borreliosis, it was found that the neutrophil/lymphocyte ratio may be a useful marker for disease activity [29]. Also, Zajkowska et al. (2011) observed that platelet parameters, such as mean platelet volume, show significant changes during the acute phase of *Borrelia burgdorferi* infection [34]. A European multicenter study confirmed that changes in the blood leukogram, although nonspecific, can contribute to the assessment of the inflammatory response and may be useful in monitoring the evolution of the disease under treatment [35]. Research continues to explore how different leukocyte subtypes and markers derived from the complete blood count can be integrated into diagnostic and prognostic algorithms for borreliosis, representing a promising area for the development of accessible clinical tools. Based on these findings, we hypothesized that the neutrophil–lymphocyte ratio, lymphocytes, monocytes, mean platelet volume, and Systemic Immuno-inflammatory Index may similarly serve as indicators of disease progression and host response in experimental Lyme borreliosis. We hypothesize that hematological markers may offer critical insights into the distinct phases of *Borrelia* infection, ranging from initial dissemination to chronic persistence, in patterns that mirror those observed in human infectious diseases. Accordingly, the present study aims to characterize the dynamics of the host response to *Borrelia bavariensis* by systematically analyzing hematological parameters as potential diagnostic biomarkers in an experimental murine model. This approach is intended to generate data of translational relevance, contributing both to the understanding of human borreliosis and, in particular, to the early confirmation of infection in the murine model; this is a prerequisite for the rational evaluation of novel therapeutic interventions.

## 2. Materials and Methods

### 2.1. Ethics Statement

This study complies with ethical standards and with the provisions of the EU Directive 63/2010 and Law 43/2014, which ensure the appropriate care, use and protection of animals involved in scientific research. Animal experiments were conducted at the Baneasa Animal Center (BAF), within the Preclinical Testing Unit of the National Military Medical Institute for Research and Development (CI) Cantacuzino, Bucharest, an authorized use unit recognized by the competent authorities. The study received favorable approval from the Ethics Committee of the CI and the Bucharest Sanitary, Veterinary and Food Safety Directorate no. 05/06.03.2023. Strict measures were implemented to minimize animal suffering during disease induction, clinical monitoring and sample collection.

### 2.2. Study Design

#### 2.2.1. *Borrelia* Strain and Genetic Modification

The *Borrelia bavariensis strain* SKT-7.1 (Serotype 4, GenBank accession number GU906888.1) was originally isolated from the cerebrospinal fluid of a human patient and kindly provided by the Institute of Neuroimmunology, Košice, Slovakia. This strain was initially designated as *Borrelia garinii* serotype 4 and later reclassified as *Borrelia bavariensis*. The bacteria were cultured in complete BSK-II medium (Sigma Aldrich, St. Louis, MO, USA) supplemented with 6% rabbit serum and incubated at 33 °C for approximately 10–14 days to reach early log phase growth. Culture purity, morphology, and motility were routinely examined under dark-field microscopy (40× magnification) (Nikon BioImaging Labs, Leiden, The Netherlands).

The SKT-7.1 strain expressing Green Fluorescent Protein (GFP) was generated as previously described [36]. Briefly, the pTM61 plasmid—harboring a constitutively expressed gfp gene under the control of the *Borrelia burgdorferi* flaB promoter (PflaB*), replication origins for both *E. coli* and *Borrelia*, and a gentamicin resistance cassette (aacC1)—was electroporated into electrocompetent SKT-7.1 cells following the standard protocol [37]. Transformants were selected in BSK-II medium containing 100 µg/mL gentamicin, and stable GFP expression was verified by epifluorescence microscopy. The strain was modified to express GFP fused to the flaB gene encoding flagellin, without affecting the motility of the spirochetes, as confirmed by dark-field microscopy.

Gentamicin was used as a selective agent in culture to maintain the plasmid carrying the gfp construct within the *Borrelia* population. This selective pressure ensures that only plasmid-bearing spirochetes persist in the culture, maintaining stable GFP expression and allowing consistent visualization of live spirochetes under fluorescence microscopy.

#### 2.2.2. Inoculum Preparation and Bacterial Quantification

The GFP-expressing *Borrelia bavariensis* strain was cultured in BSK-H medium supplemented with 6% rabbit serum and 100 µg/mL gentamicin at 33 °C for approximately 10 days. Cultures were monitored every 4 days using both dark-field and fluorescence microscopy until maximal growth was reached. Bacterial concentration was determined with a Petroff–Hausser counting chamber (Hausser Scientific, Horsham, PA, USA), and to obtain the desired inoculum, cultures were centrifuged at 3500× *g* for 10 min. The pellet was resuspended in fresh BSK-H medium to achieve a final concentration of 5 × 10^6^ spirochetes/mL, verified by a second count. The inoculum was prepared from low-passage cultures (<5 passages) to preserve infectivity.

This approach ensures reproducibility and allows direct monitoring of spirochete distribution in host tissues under fluorescence microscopy, as GFP expression enables in situ visualization during infection and treatment studies [38].

#### 2.2.3. Animal Inoculation Technique

The animals were fully anesthetized with a mixture of Xylazine (3 mg/kg, Farmavet, Bucharest, Romania) and Ketamine (50 mg/kg, Biotur, Alexandria, Romania), intraperitoneally administered, with the dose being dependent on individual weight, followed by retro-orbital blood collection into EDTA-coated vacutainers for hematological examination. Preparation for spirochete inoculation included shaving the fur in the dorsal thoracic region and disinfecting the skin with 70% alcohol solution and 3% iodine. Under a laminar flow hood, 100 µL (respectively, a concentration of 5 × 10^5^ spirochetes/mouse) of suspension was injected intradermally at approximately 10 sites, in order to ensure a uniform distribution of spirochetes in the dermal tissue and to better reproduce the multifocal pattern of natural tick-borne transmission. This approach increases the likelihood of consistent infection and effective dissemination throughout the host tissues while minimizing inter-individual variability between animals. After this procedure, the animals were placed in clean bedding boxes to recover from anesthesia.

**Figure 1:**
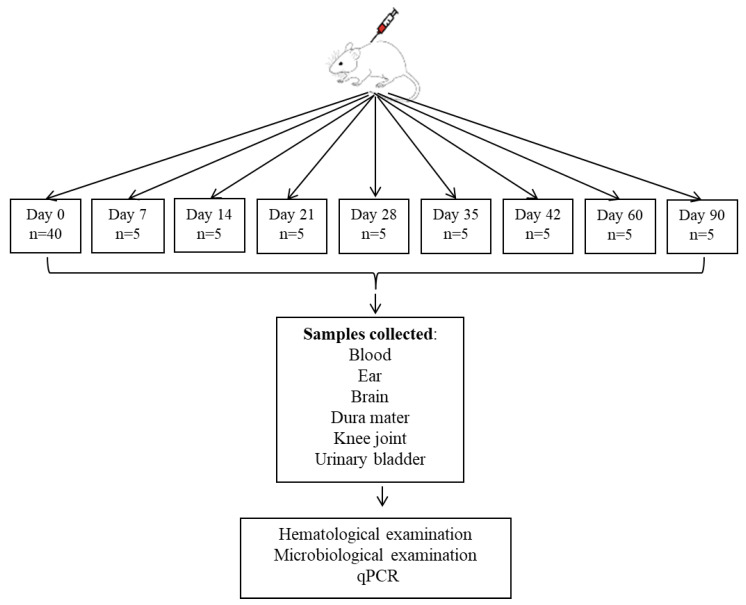
Diagram of the experimental study showing the stages of the study, where day 0 represents the moment of intradermal inoculation with the *Borrelia* strain, and the following days represent the intervention points for sample collection. n = number of animals used.

The monitoring period lasted 90 days, and blood samples were collected at predefined time intervals. Additionally, five mice were euthanized at each interval using an anesthetic overdose (150 mg/kg Ketamine), and samples were collected for microbiological analysis, or qPCR (Figure 1).

#### 2.2.4. The Hematological Analysis

The hematological examination was performed on venous blood samples collected at predetermined intervals, and processed using the IDEXX ProCyte Dx analyzer. To achieve the study’s objective, the following white blood cells were analyzed: neutrophil–lymphocyte ratio, lymphocytes, monocytes, mean platelet volume, and Systemic Immuno-inflammatory Index (SII), which was calculated using the formula: SII = (NEU count × PLT (platelet count)/LYM count.

Before the end of each period (days 7, 14, 21, 28, 35, 42, 60, and 90), the mice were injected intraperitoneally daily with gentamicin (1 mg/kg) to enhance the visualization of *Borrelia* under fluorescence microscopy. Brain, dura mater, knee joint, urinary bladder, and ear samples were collected from the euthanized animals, and divided into tubes based on the specific analysis required: brain and dura mater were placed together in a tube (hereafter referred to as the “head”), while the knee joint, ear, and urinary bladder were collected in a separate tube (hereafter referred to as the “body”).

#### 2.2.5. The Microbiological Analysis

For the microbiological analysis of the tissues a modified BSK-H medium (BSK-H with antibiotics) was used which contained gentamicin 100 µg/mL (to stimulate the GFP expression in the bacterial strain and as a selective component against potential bacterial contaminants), and amphotericin B 2.5 µg/mL (as a selective component against potential fungal contaminants). The samples were incubated at 33 °C and checked every 4 days for the presence of spirochetes by fluorescence microscopy.

#### 2.2.6. DNA Extraction and Quantitative PCR Analysis

The samples intended for qPCR analysis were stored at −80 °C in cryotubes containing glycerol and Brain Heart Infusion (BHI) medium (Oxoid, UK) in a 1:1 ratio until testing, which was conducted at the Histovet Laboratory (Bucharest, Romania).

DNA extraction and quantitative PCR (qPCR) for the detection of *Borrelia bavariensis* DNA were performed by Histovet using their standardized and validated diagnostic protocol. Briefly, total DNA was extracted from collected tissue samples (ear, dura mater, brain, bladder and joint) using a silica-column-based commercial kit (Qiagen DNeasy Blood & Tissue Kit or equivalent) (Qiagen, Venlo, The Netherlands), following the manufacturer’s recommendations.

Detection and quantification of *Borrelia* DNA were performed by real-time PCR targeting the flaB gene, following previously described methods [39]. All reactions were carried out in triplicate, and negative controls were included in each run. The results were expressed as the number of *Borrelia* genome equivalents per microgram of total DNA.

### 2.3. Animal Selection

The study involved the use of 40 C3He/HeNClr mice, males, aged 12–14 weeks, obtained from the Specific Pathogen Free animal breeding facility (BAF) of CI. All experimental procedures followed the provisions of international guidelines as stipulated in “The ARRIVE Guidelines 2.0: Author Checklist—The ARRIVE Essential 10” [40]. The animals were housed in the experimental facility in individually ventilated cages (Tecniplast, Buguggiate, Italy), with ad libitum access to food and water. The microclimate conditions included 12 h light/dark cycles, a temperature of 20–24 °C, and 40–60% relative humidity. The acclimatization period lasted 5 days.

### 2.4. Statistical Analysis

The number of animals used in this study represents the statistical minimum necessary to ensure the validation of the studies. To determine the number of mice required, we used the power analysis method using IBM SPSS Statistics software 31.0.1.0. GraphPad Prism 10.4.1 software (La Jolla, CA, USA) was used to calculate the sample size, setting the α error at 0.05 and the power at 80%. The Shapiro–Wilk test was applied to verify the normality of the data distribution for all parameters studied. For parameters with a normal distribution, differences in white blood cell counts were analyzed using the one-way ANOVA test. Post hoc comparisons were performed using the Bonferroni test when equal variances were assumed. For parameters with a non-normal distribution, non-parametric tests were applied (Kruskal–Wallis test followed by multiple comparisons using the Dunn test). The results for these parameters are expressed as median and interquartile range. Results for normally distributed parameters are expressed as mean ± standard error of the mean (SE), because SE provides an estimate of the precision of the sample mean as an estimator of the population mean, being more relevant for comparisons between groups in the context of our experimental study. All comparisons were performed at a significance level of *p* < 0.05.

### 2.5. Generative AI Tools

(OpenAI’s ChatGPT, GPT-4) were used to assist in rephrasing sentences for clarity, checking grammatical errors, and drafting the graphical abstract. All content generated by AI was carefully reviewed, edited, and verified by the authors to ensure scientific accuracy and integrity.

## 3. Results

### 3.1. Microbiological Examination and qPCR Analysis

Microbiological examination and qPCR revealed that samples were positive from day 7 to day 21, with negative results on days 28–42, and on days 60 and 90, *Borrelia* could only be detected by qPCR analysis (Table 1).

### 3.2. Hematological Examination

To analyze the correlation between hematological parameters and immune response in *Borrelia bavariensis* infection, several aspects related to the number of neutrophils, lymphocytes, monocytes, basophils and the systemic immuno-inflammatory index must be taken into account.

**Figure 2:**
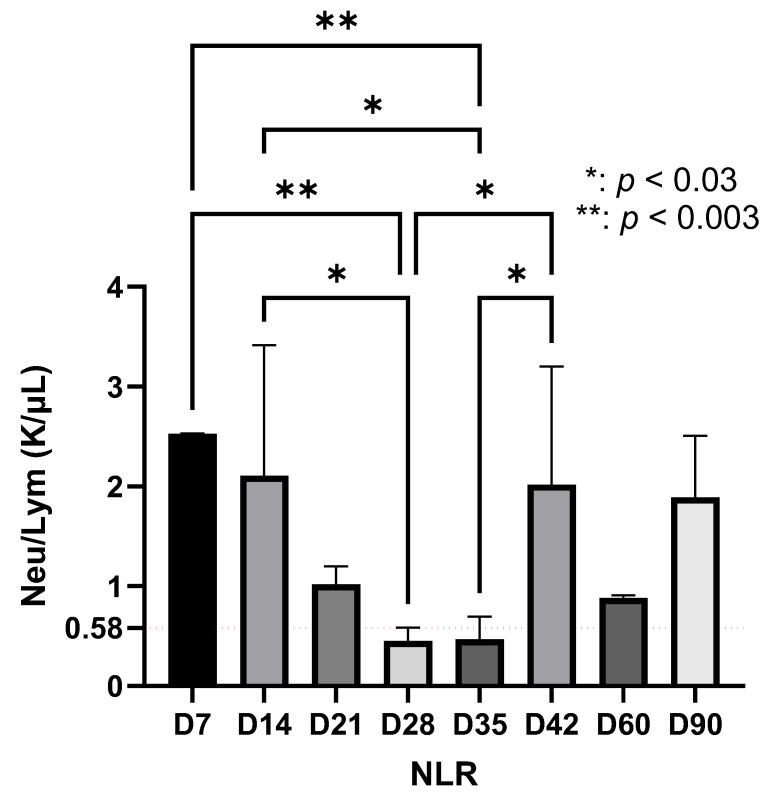
NLR during the study (red line represents the mean value of neutrophils at day 0). Neutrophil dynamics showing two peaks, one at day 7 (~2.52 K/μL) and the second at day 42, with a decrease between the two peaks at day 35 (*p* < 0.003). After the peak at day 42 (~2.01 K/μL), NLR decreased sharply on day 60, then increased slightly until day 90 (Statistical significance was determined using one-way ANOVA, K/µL = 10^3^ cells/µL).

The NLR showed a dynamic evolution during the experimental period. In the early stage, the NLR values were elevated. Between days 21 and 35, a marked decrease in NLR was recorded, followed by a subsequent increase after day 35, which persisted until the end of the observation period (day 90) (Figure 2).

**Figure 3:**
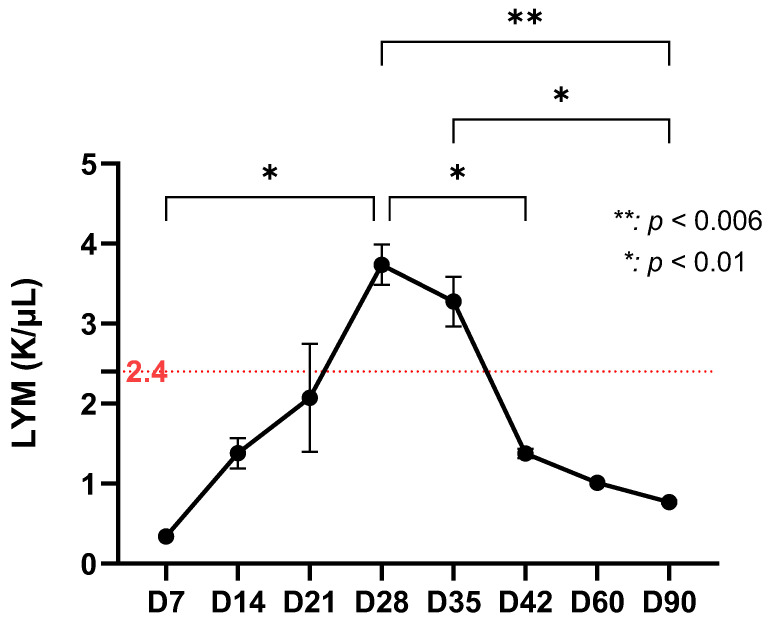
Lymphocyte dynamics in the experimental study (red dotted line representing the average lymphocyte value from day 0) suggest a progressive increase from day 7 to a peak on day 28 (~3.7 K/μL) followed by maintenance of a high level on day 35 (~3.3 K/μL) and then a sharp decrease on day 42 (~1.3 K/μL). By the end of the study (day 90), lymphocytes reach values below 1 K/μL.

Lymphocytes, which are essential in generating the adaptive immune response, showed a significant increase on day 28 and day 35 (*p* < 0.01). However, their number then began to decline continuously, with a sharp decrease on day 42 and a relatively constant level thereafter, up to day 90 (*p* < 0.006) (Figure 3). Lymphocytes mark the transition to adaptive immunity. The peak at D28–D35 corresponds to the initial bacterial clearance, while the subsequent decline suggests stabilization of the immune response.

**Figure 4:**
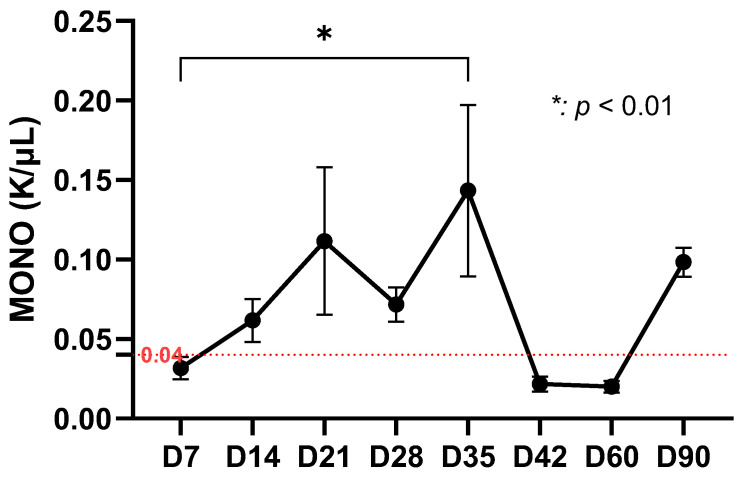
Monocyte dynamics in the experimental study (red dotted line representing the average value of monocytes from day 0) shows low values on day 7, similar to basal level, fluctuating between days 21–28, with a pronounced peak on day 35 (~0.15 K/μL) followed by a significant decrease on days 42 and 60 (0.02 K/μL), then a significant increase on day 90 (~0.10 K/μL).

Monocyte counts showed a gradual increase from the initial value of 0.04 K/µL, reaching their highest levels on day 21, followed by statistically significant fluctuations (*p* < 0.01) until day 35. After day 42, monocyte values decreased and remained stable until the end of the observation period (Figure 4).

**Figure 5:**
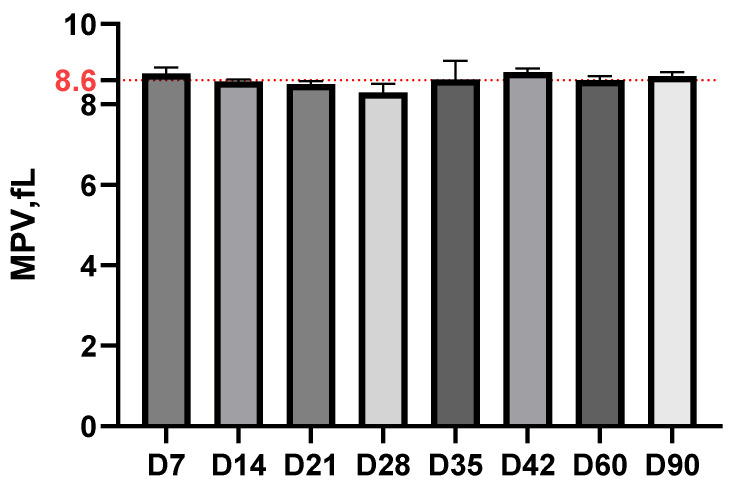
MPV dynamics in the experimental study (red dotted line representing the average value of MPV from day 0).

The mean platelet volume (MPV) remained remarkably stable, around 8.6 fL throughout the experiment, without significant variations between different time intervals, suggesting that *Borrelia* infection did not significantly influence platelet morphology and functionality (Figure 5).

**Figure 6:**
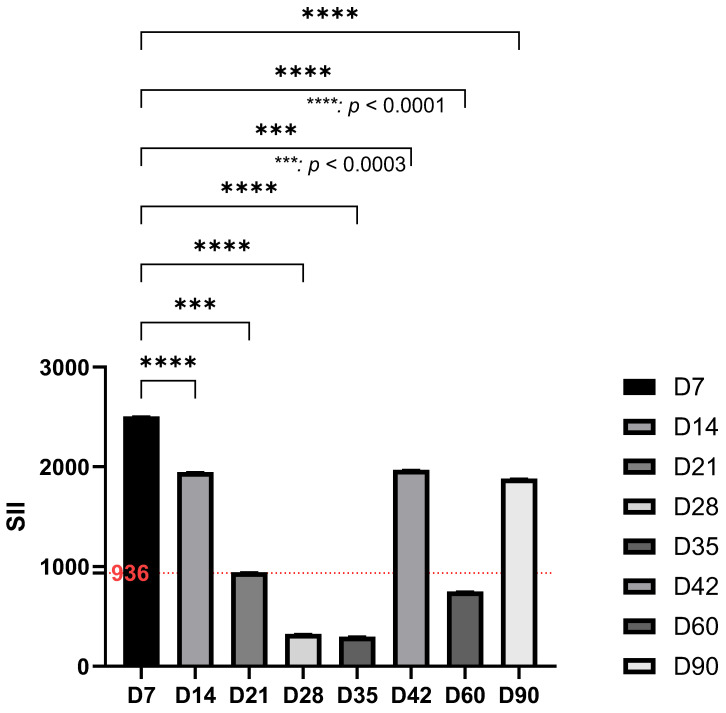
Systemic immuno-inflammatory index calculated during the study reaches a significant peak at day 7 compared to basal values (dotted red line), followed by a decrease until day 35, followed by an increase at day 60 and 90.

The SII showed dynamic changes during the experimental period. A marked peak was recorded on day 7 (*p* < 0.0001), followed by a progressive decrease until day 35. A secondary increase was observed on day 42, with subsequent fluctuations that persisted until the end of the study (Figure 6).

Integrative interpretation of the results indicates that in experimental infection with *Borrelia bavariensis*, in mice, there are 3 phases of evolution and immunological response that can be observed by analysis of the blood count, as summarized in Table 2. This detailed analysis demonstrates the complexity of the immune response in infection with *Borrelia bavariensis* and the importance of monitoring hematological parameters for understanding the host–pathogen interaction.

## 4. Discussion

The aim of the study was to evaluate the utility of hematological parameters (NLR, LYM, MONO, MPV and SII) as potential immuno-inflammatory markers for monitoring the progression of borreliosis in an experimental mouse model. This approach represents a novel and non-invasive method for tracking the infection and provides valuable information on the dynamics and efficiency of the host immune response. Analysis of the complete blood count may allow prediction of bacterial persistence or reactivation. To confirm this hypothesis, future studies using RNA-based qPCR or transcriptomic analyses could help determine whether the detected bacteria exhibit metabolic activity.

*Borrelia bavariensis* has a strong tropism for human infection, often causing severe neuroborreliosis [41] and is also well adapted to rodent hosts [8,42]. *Borrelia* species employ complex immune evasion strategies that allow survival in both the bloodstream and deep tissues [43]. Consequently, host-mediated inflammatory reactions, rather than direct bacterial damage, appear to be responsible for tissue pathology.

*Borrelia bavariensis* is a species of the Bbsl complex that can cause LD, and factors involved in the pathogenesis of the disease can be studied using animal models. Although animals do not fully reproduce human borreliosis, they are useful in understanding the mechanisms related to invasion, dissemination and persistence. The different immune responses generated by the host in response to *Borrelia* make understanding the pathogenesis of the disease extremely complex. SII provides an integrated reflection of the balance between the innate and adaptive arms of immunity. Its correlation with the dynamics of neutrophils, lymphocytes and platelets makes it a sensitive indicator of systemic inflammation and immune activation. The fluctuations observed during infection highlight the alternating predominance of innate and adaptive responses, supporting the idea that SII can serve as a surrogate marker of the inflammatory burden and the intensity of the immune response in experimental borreliosis, as has been observed in other conditions such as cancer, pancreatitis or other inflammatory diseases [44,45,46]. In our study, the immune response was triggered on day 7, with a remarkable involvement of NLR and SII. This finding could suggest an attempt by *Borrelia bavariensis* to evade the host immune response in its effort to disseminate to organs and tissues. Furthermore, the significant increase in monocytes and lymphocytes, accompanied by a reduction in the systemic immunoinflammatory index between days 28 and 35 of the experiment, indicates the activation of the adaptive immune response and the generation of anti-*Borrelia* antibodies. This can be correlated with negative results from both microbiological and qPCR examinations.

Negative qPCR results at day 35, followed by positivity at later time points (days 60 and 90), could reflect temporary reductions in bacterial numbers below the detection limit or transient transitions into non-culturable, metabolically inactive forms. In *Borrelia* species, attenuation of infectivity during culture is often linked to the loss of plasmids required for persistence in natural hosts [43]. Negative microbiological results, in parallel with qPCR positivity, therefore suggest either temporary bacterial clearance or persistence in latent, non-culturable states. It is important to note that qPCR detects bacterial DNA regardless of bacterial viability, making it impossible to distinguish between live and dead organisms. The reappearance of qPCR positivity at later stages is consistent with reports describing the transformation of *Borrelia* into spherical or persistent L-forms under stress conditions, such as antibiotic exposure or immune pressure [47,48]. These metabolically inactive or slow-growing forms may undergo surface protein modifications that prevent immune recognition and culture detection yet remain viable and capable of reverting to the motile spirochetal form when conditions permit [49]. The persistence of bacterial DNA along with immuno-inflammatory activity suggests a continued host–pathogen interaction even in the absence of culturable organisms.

Because hematological assays are widely available, our findings highlight their potential as valuable, low-cost indicators for studying the persistence and immune dynamics of *Borrelia* in experimental settings. The observed immune oscillations indicate that *Borrelia* can reactivate even after apparent clearance, a phenomenon also supported by previous studies demonstrating long-term persistence in host tissues [50].

In natural infections, *Borrelia* are inoculated into the skin (epidermis or dermis) by ticks, with the depth of inoculation depending on the size of the tick and the thickness of the host skin. Studies have shown that experimental intradermal inoculation with different concentrations of *Borrelia* reproduces tissue burdens similar to those observed in natural tick-borne transmission, with variations depending on the mouse strain [51].

In our study, we chose intradermal inoculation of C3HeNcrl mice, a commonly used model for studying this disease, because it closely mimics natural infection, allows spirochetes to migrate rapidly through the dermis via collagen fibers [52], and facilitates efficient dissemination into collagen-rich tissues [24]. *Borrelia* viability was confirmed by positive cultures from samples collected from the ear, bladder, and joints. Although some spirochetes enter the bloodstream—as evidenced by leukocyte responses—the majority migrate through the dermis and lymphatic system [53]. A relevant example is the dissemination of *Borrelia* from the inoculated ear to the contralateral ear, a process that takes approximately eight days [54]. This observation explains the possible false-negative culture results (when samples are taken from areas where *Borrelia* have not yet reached) or delayed culture positivity, phenomena also observed in our study.

Infection control involves both innate and adaptive immune mechanisms, with a special role for the humoral response in reducing bacterial tissue burden, resolving clinical manifestations, and providing protection against reinfection. Innate immune mechanisms remain active throughout the course of infection, but are particularly important in the early stages. This complexity of the immune response highlights the importance of the murine immune system in defending against infections and developing protective immunological memory [55]. Through this study, we demonstrated that the immune response to *Borrelia bavariensis* exposure can also be assessed in the late stages of infection by analyzing blood parameters obtained from complete blood counts. Detection of *Borrelia* only by qPCR on days 60 and 90 could indicate the presence of bacterial DNA in tissues or possible transformation of bacteria into unculturable forms, which may contribute to inflammatory processes, as suggested by the SII results observed on day 90, as well as bacterial persistence and development of immunological memory.

This study has some methodological limitations, in particular the absence of quantitative determinations of proinflammatory cytokines and specific anti-*Borrelia* antibodies. In addition, our research lacks the analysis of selected cytokines and chemokines by multiplex assays, the correlation of hematological parameters with specific molecular markers, and the interpretation of the results in the context of known immune signaling pathways in Laryngeal disease. To validate and extend the current results, future research will aim to incorporate these immunological parameters into the experimental protocol. We intend to implement multiplex assays for key cytokines and chemokines, establish correlations between hematological findings and molecular immune markers, and provide a more comprehensive interpretation within the framework of established immune signaling pathways in Lycopersicum disease. In addition, we intend to evaluate immunological changes in the context of standard antimicrobial therapy to characterize the dynamics of the immune response under conditions of *Borrelia* clearance.

This research makes original contributions by implementing a new methodology for assessing the immune response to *Borrelia bavariensis* infection in a murine model, representing the first documentation of the pathogenicity of this specific strain in an experimental mouse model. In addition, our findings contribute to the development of a diagnostic approach with potential translational applications in clinical practice. The applicability of this methodology in human medicine offers the advantage of significantly reducing the costs associated with both the initial diagnosis of LD and the post-treatment monitoring of spirochete clearance. This optimization of the diagnostic process could have important implications for the clinical management of patients.

## 5. Conclusions

The integration of hematological findings with direct diagnostic methods (culture, qPCR) confirms the value of complete blood count (CBC) parameters as accessible biomarkers for monitoring the course of *Borrelia bavariensis* infection. This approach enabled the identification of three distinct evolutionary and immunological phases of murine borreliosis, thereby contributing to a more refined understanding of host–pathogen dynamics. Both the SII and the NLR exhibited a pronounced peak on day 7 post-infection, followed by a progressive decline, findings that were consistent with microbiological and molecular results. These observations demonstrate that CBC-derived indices can serve as reliable markers for disease progression, immune response monitoring, and the identification of critical inflection points in the infection’s trajectory. Importantly, by establishing the translational potential of such simple hematological analyses, this study supports their applicability not only for experimental confirmation of infection in murine models used to test novel therapies but also as a basis for developing early and practical diagnostic approaches in human borreliosis.

## Figures and Tables

**Table 1:** Compilation of the results from microbiological examinations (*Borrelia* culture in BSK-H medium and incubation) and qPCR analysis.

Group Analyzed on Euthanasia Day	Microbiological Examination (Culture)	qPCR
7	+ (head)	+ (head and body)
14	+ (head)	+ (head and body)
21	+ (body)	+ (body)
28	-	-
35	-	-
42	-	-
60	-	+ (head and body)
90	-	+ (head and body)

**Table 2:** Correlation between phases of infection and immune response.

The Evolutionary Phase of Borreliosis	Immunological Characteristics
Early phase (D0–D14)	Positive detection of *Borrelia* by both methods (culture and qPCR)
	Significant increase in NLR at D7
	Maximum systemic immuno-inflammatory index at D7 (*p* < 0.0001)
	Dominant innate immune response, reflected by neutrophil mobilization
	This phase corresponds to the period of active dissemination of spirochetes
Intermediate phase (D14–D35)	Presence of *Borrelia* confirmed until D21, then negative
	Significant increase in lymphocytes (*p* < 0.01) at D28–D35 Increase in monocytes with peak at D35 (*p* < 0.01) Decrease in systemic immuno-inflammatory index Transition to adaptive immune response
Late phase (D35–D90)	Detection of *Borrelia* only by qPCR at D60 and D90 NLR reactivation at D42 (*p* < 0.03) Decrease and stabilization of lymphocytes after D42 Fluctuations in the immuno-inflammatory index Suggests bacterial persistence and development of immunological memory

## Data Availability

The original contributions presented in this study are included in the article. Further inquiries can be directed to the corresponding authors.

## Abbreviations

The following abbreviations are used in this manuscript:

SII	Systemic immuno-inflammatory index
CBC	Complete blood count
IFN-γ	Interferon-gammaNF
Ig	Immunoglobulin
DNA	Deoxyribonucleic acid
BAF	Băneasa Animal Facility
CI	Cantacuzino National Military Medical Institute for Research and Development
GFP	Green Fluorescence Protein
EDTA	Ethylenediaminetetraacetic acid
qPCR	Quantitative polymerase chain reaction
NEU	Neutrophils
LYM	Lymphocytes
MONO	Monocytes
BASO	Basophils
PLT	Platelet count
